# Chronic stress and turnover intention of resident physicians after experiencing COVID-19

**DOI:** 10.1186/s12909-023-04681-8

**Published:** 2023-09-27

**Authors:** Qingwen Jia, Yi Qu, Huisheng Huo, Hongxia Yin, Meijun Jiang, Dianping You

**Affiliations:** 1 Organization and Personnel Department, Children’s Hospital of Hebei Province, Shijiazhuang, China; 2 Scientific research division, Children’s Hospital of Hebei Province, Shijiazhuang, China; 3 Human resources department, Affiliated hospital of north Sichuan medical college, Nanchong, China; 4 Graduate School, Hebei Medical University, Shijiazhuang, China; 5 Party and Government Integrated Office, Children’s Hospital of Hebei Province, Shijiazhuang, China

**Keywords:** COVID-19, Resident physicians, Stress, Chronic stress, Turnover intention

## Abstract

**Background:**

Previous studies have explored the stress and turnover intention of healthcare workers, but as important backup talents in the healthcare system, resident physicians have received little attention from researchers, especially after experiencing COVID-19. Therefore, this study aims to evaluate the chronic stress and turnover intention of resident physicians after experiencing COVID-19.

**Methods:**

From June to August 2022, we conducted a questionnaire survey on resident physicians in the Children's Hospital of Hebei Province through the online platform (Wenjuanxing) to evaluate their chronic stress and turnover intention after experiencing COVID-19. For the collected data, we used frequency and percentage to make the statistical description, the Chi-square test to make a univariate analysis on the scores of chronic stress and turnover intention scale, and binary logistic regression analysis to explore the influencing factors of turnover intention.

**Results:**

Out of 143 respondents, we finally received 127 questionnaires, with a response rate of 88.81%. Among 127 respondents, 80.31% of resident physicians experienced varying degrees of chronic stress (mild: 36.22%, moderate: 35.43%, severe: 8.66%), and 74.80% of resident physicians showed varying degrees of turnover intention (mild: 23.62%, moderate: 37.79%, severe: 13.39%). Moreover, age (OR = 0.772, *P* = 0.042), identity (OR = 8.648, *P* = 0.021), and chronic stress levels (mild: OR = 6.938, *P* = 0.003; moderate: OR = 44.049, *P* < 0.003; severe: OR = 46.141, *P* = 0.004) can significantly affect turnover intention.

**Conclusion:**

In this study, we reported a relatively high proportion of resident physicians with high chronic stress and high turnover intention after experiencing COVID-19. We suggest that the relevant departments should pay more attention to the resident physicians’ group and formulate corresponding measures to solve the problems faced by the resident physicians and ensure the stability of the health human resources.

## Introduction

Scholars have various definitions of stress. Some scholars defined stress as a specific physiological response triggered by the body in the face of unpredictable or uncontrollable demands (i.e., threats or challenges) [1, 2]. Other researchers think that stress is a situation where the need exceeds the individual's ability to respond. It is generally a combination of two elements; Unable to meet the proposed requirements or the judgment made by the individual that the requirements cannot be met [3, 4]. Stress can be subdivided into acute and chronic [5, 6]. Although the definition and translation of chronic stress are different, chronic stress usually refers to "abnormal" stress that lasts or lasts for a long time, either because it occurs repeatedly, intermittently, and continuously, or because it constitutes a serious threat, rather than easy to adapt or overcome. It refers to background or environmental stress due to more or less constant stress sources embedded in the living or working environment [7]. That is, chronic stress is conceptualized as the sum of long-term exposure to one repeated or multiple acute stress sources [1, 8].

The negative effects of stress cannot be ignored. Although acute stress can even promote short-term adaptation by mobilizing resources, chronic stress seems to have only destructive effects [9]. Chronic stress is considered to be an important risk factor for physical and mental health, and its negative impact on health is more obvious than the experience of a single stress event [10]. Many adverse physiological reactions and diseases have been proven to be associated with chronic stress, such as researchers finding that caregivers with high levels of stress are more likely to feel depressed, which can further lead to unhealthy dietary behaviors [11]. Similarly, researchers found that atherosclerotic cardiocerebrovascular disease is usually caused by chronic stress [12]. In addition, chronic stress can not only lead to adverse physiological reactions but also lead to many adverse psychological problems. Previous studies have confirmed that stress can hurt the lives of medical students and may lead to depression and suicide [13]. Moreover, chronic stress also seems to affect people's attitudes towards and choice of occupation. Zhang et al. found that perceived stress can affect professional identity, and the professional identity of nurses decreases with increasing stress [14]. And Nashwan et al. found that during COVID-19, the stress level of nurses played an important role in turnover intention [15].

The medical system had always been considered to be under chronic stress. Studies had shown that the chronic stress level of medical personnel is higher than that of the general population [16, 17] because they are more likely to be exposed to the risk factors leading to chronic stress, such as high workload, night shift, and staff shortage [8, 18]. Similarly, medical students are more susceptible to stress than students in other disciplines because they face many academic challenges [19], such as interviews, exams, and internships [13]. The tense learning environment, heavy academic tasks, and high academic performance are all important sources of stress [20]. However, compared with them, the plight faced by resident physicians undergoing training is more worrying. Resident physicians have the dual conditions of medical students and attending doctors. As novices in clinical work, resident physicians bear many responsibilities but have low welfare remuneration [21, 22]. Under various factors, some researchers regard the professional clinical training of doctors as a natural chronic stress paradigm [23]. Worse still, COVID-19 had affected the medical system and brought unprecedented challenges to medical personnel [24–26]. The persistent COVID-19 had been described as a widespread chronic stressor that affects people and all social strata around the world [8, 27, 28]. In this serious public health problem, resident physicians have to contact patients with COVID-19 directly [29]. COVID-19 has brought unprecedented pressure to resident physicians, including but not limited to increased workload, unsafe work, and insufficient personal protective equipment (PPE) [29–31]. In addition, in addition to work-related pressures, resident physicians must also adapt to significant changes in education, such as restricted training, which may delay their career development [31].

Turnover intention is the main predictor of resignation behavior, which refers to employees’ intention and willingness to resign. It can predict the actual resignation behavior and is considered the strongest predictor of the actual resignation of medical personnel [24, 32]. Training doctors is an expensive and long process, and the loss of well-trained doctors and medical workers will lead to a significant loss of human capital [33]. At the same time, a large-scale, multi-institutional study conducted in China had shown that the turnover intention level of the students who participate in the standardized resident training program is high, and as high as 47.87% of the resident physicians show a "high" or "very high" turnover intention level [34]. This is undoubtedly bad news because resident physicians are an important way for medical students to become doctors. Due to the lengthy education and training required for doctors, a higher turnover intention can increase the shortage of medical resources and damage patient satisfaction [35, 36]. And resignation will consume human resources and incur financial costs for hiring and training new employees [37, 38]. Finally, a higher turnover intention can lead to an increase in the workload of the remaining personnel, leading to a "vicious cycle" of personnel turnover and exacerbating the crisis of health human resources [39].

In such a special period as COVID-19, we must focus on resident physicians, because most of the research focuses on medical personnel and medical students, and focuses on topics such as mental health, professional identity, turnover intention, whether they have participated in the front-line work of COVID-19 or not [24, 25]. Few studies have focused on the special group of resident physicians. At the same time, research on resident physicians mainly focuses on mental health, such as anxiety, depression, and burnout [40]. The assessment of resident physicians' stress focuses more on acute stress rather than more damaging chronic stress. In addition, most studies have mainly explored the influencing factors of stress among resident physicians during COVID-19, while few studies have explored the harm of resident stress perception on medical human resources during COVID-19. Therefore, we conducted this study to evaluate the chronic stress and turnover intention of resident physicians after experiencing COVID-19.

## Materials and methods

### Study participants and design

This study is a cross-sectional study conducted at the Children's Hospital of Hebei Province, using the Wenjuanxing (www.wjx.cn). This hospital is the only provincial-level children's hospital in Hebei Province, China. It is also a standardized training base for pediatric resident physicians in Hebei Province, undertaking the task of training graduate students and resident physicians. There were 143 people in the base, including graduate trainees and non-graduate trainees participating in resident training in the standardized training base. The questionnaire was distributed to resident physicians by department staff in charge of standardized training for resident physicians between June and August 2022. Links to the questionnaire were distributed through WeChat to ensure that every respondent had the opportunity to answer our questions.

This project collects resident physicians' feelings about standardized training and their career choices after undergoing standardized training. This study is part of it, including the following three points:(I) basic personal information, (II) perceived chronic stress, and (III) possible turnover intention. The respondents were asked to carefully answer some relevant questions to understand their thoughts on each subject.

This study was conducted anonymously, and each researcher voluntarily participated in this study. As the first question in the questionnaire, the researcher must express informed consent before continuing to answer the follow-up questions. In the process of completing the questionnaire, the respondents could contact us if they have any questions. At the same time, this study was approved by the medical ethics committee of Hebei Children's Hospital. In the end, out of 143 resident physicians, we received a total of 127 questionnaires, with a response rate of 88.81%.

### Measures

#### Chronic stress

We used the nine-item short form of the Trier Inventory for Chronic Stress Scale(TICS-9) to measure chronic stress faced by respondents [41]. The original Trier Inventory for Chronic Stress Scale(TICS-57) is a standardized German scale with 57 items, including 9 content dimensions: Work Overload, Social Overload, Pressure to Perform, Work Discontent, Excessive Demands from Work, Lack of Social Recognition, Social Tensions, Social Isolation, and Chronic Worrying [42]. The TICS-57 was translated into English by Petrowski et al. and the TICS-9 was developed by selecting an item from each content dimension. Their result had shown that the TICS-9 shows a very good factorial structure, has a high correlation with the original version, provides all the validity of the full-length form, and is suitable for large multivariable research. Cronbach's alpha is 0.880 [43, 44]. The TICS-9 used Likert's 5-point scoring method (1 = Never to 5 = Very often) with the total score being between 9 and 45. The higher the score means the higher the perceived chronic stress. Meanwhile, based on the threshold classification of similar scales by previous researchers, we classified the TICS-9 into normal, mild, moderate, and severe chronic stress (normal: 9–17; mild: 18–26; moderate: 27–35; severe: 36–45). In this study, the Cronbach's alpha of TICS-9 is 0.959, the KMO value is 0.905, and the *p*-value of Bartlett's test is less than 0.001. At the same time, when using the maximum variance method for principal component analysis, only one common factor is extracted, with a cumulative percentage of variance of 75.608%.

#### Turnover intention

The Turnover Intention Questionnaire(TIQ) formed by Camman et al. was modified and applied to measure the turnover intention of the respondents [45]. We rewrote the items in the questionnaire by replacing the subject in the original questionnaire, making turnover intention relevant to our research [46]. Specifically, in the first term, "medical profession" was replaced with "pediatric course"; The 'has zero contact with patients' in the second term was rewritten as' zero contact with pediatrics'; The 'medical course' in the third term was rewritten as' pediatric profession'. Finally, the turnover intention was assessed by three items, “If I could choose again, I would choose not to be on the pediatric course”, “It is very possible that I will look for a career in the healthcare industry that has zero contact with pediatrics in the future”, “I often think of not going into the pediatric profession in the future”. The TIQ used Likert's 5-point scoring method (1 = Extremely disagree to 5 = Extremely agree). The total score is between 3 and 15. The higher the score means the higher the turnover intention. Meanwhile, based on the threshold classification of similar scales by previous researchers, we classified the TIQ into normal, mild, moderate, and severe turnover intention (normal: 3–5; mild: 6–8; moderate: 9–11; severe: 12–15). In this study, the Cronbach's alpha of TIQ is 0.944, the KMO value is 0.754, and the *p*-value of Bartlett's test is less than 0.001. At the same time, when using the maximum variance method for principal component analysis, only one common factor is extracted, with a cumulative percentage of variance of 89.940%.

### Statistical analyses

All analyses were performed using Statistical Package for Social Sciences (SPSS 22.0). Frequency and percentage methods were used to describe the socio-demographic characteristics of the respondents participating in this study, such as gender, marital status, habitation, etc. Because of the non-normality of age, chronic stress scale score, and turnover intention scale score, the median and interquartile ranges were used to describe the concentration and dispersion of data. In addition, the Chi-square test was used for univariate analysis to evaluate the differences in the scores of the TICS-9 and TIQ in their respective variables. Finally, binary logistic regression analysis was used to find out the relevant factors affecting turnover intention. A value of *P* < 0.05 (two-tailed) was considered statistically significant.

## Results

### Socio-demographic characteristics

The age of 127 respondents is relatively concentrated. The median age is 27 years old, and the interquartile range was 25 to 29. And the distribution of resident physicians in the training years is relatively average, 33.07% in the first year, 35.43% in the second year, and 31.50% in the third year. The respondents were mainly female (78.74%), unmarried (69.29%), rural (61.42%), graduate trainees (62.20%), those without clinical experience (67.72%), and trainees with a monthly family income of 5000 yuan or less (53.54%). Table 1 shows the socio-demographic characteristics of the respondents.

**Table 1:** Socio-demographic characteristics and univariate analysis of scores on the TICS-9

Variables		Total N(%)	TICS-9		*Chi*^*2*^*-value*	*P-value*
			Normal	Chronic stress		
Gender	Male	27(21.26%)	7(25.93%)	20(74.07%)	0.845	0.358
	Female	100 (78.74%)	18 (18.00%)	82 (82.00%)		
Marital status	Unmarried	88 (69.29%)	18 (20.45%)	70 (79.55%)	0.107	0.743
	Married	39 (30.71%)	7 (17.95%)	32 (82.05%)		
Habitation	Rural	78 (61.42%)	12 (15.38%)	66 (84.62%)	2.365	0.124
	Urban	49 (38.58%)	13 (26.53%)	36 (73.46%)		
Household monthly income (CNY)	≤ 5000	68 (53.54%)	11 (16.18%)	57 (83.82%)	1.140	0.286
	>5000	59 (46.46%)	14 (23.73%)	45 (76.27%)		
Identity	Graduate trainees	79 (62.20%)	17 (21.52%)	62 (78.48%)	0.445	0.505
	Non-graduate trainees	48 (37.80%)	8 (16.67%)	40 (83.33%)		
Year of training	1st	42 (33.07%)	7 (16.67%)	35 (83.33%)	3.959	0.138
	2nd	45 (35.43%)	13 (28.89%)	32 (71.11%)		
	3rd	40 (31.50%)	5 (12.50%)	35 (87.50%)		
Clinical experience before training	No	86 (67.72%)	17 (19.77%)	69 (80.23%)	0.001	0.973
	Yes	41 (32.28%)	8 (19.51%)	33 (80.49%)		

### Chronic stress

**Fig. 1:**
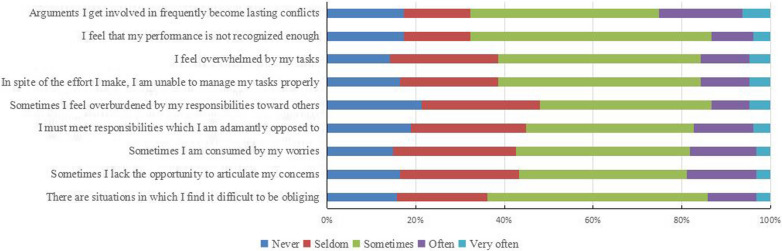
Details of each item on TICS-9

**Fig. 2:**
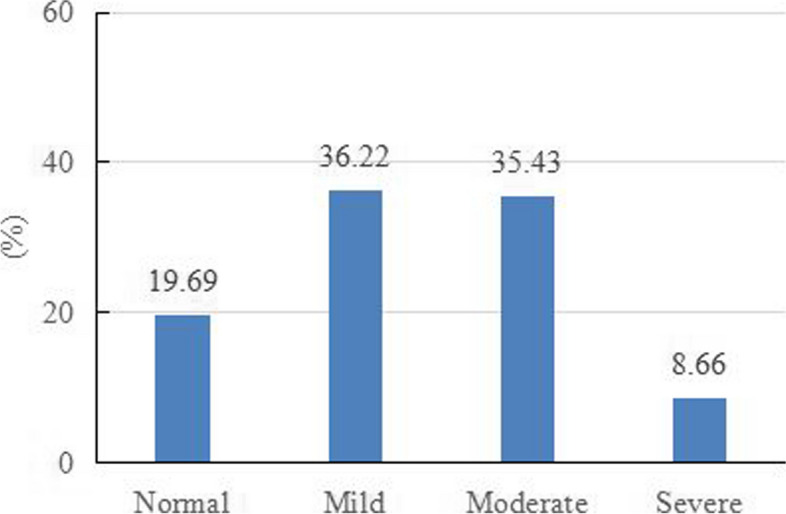
Proportion of respondents with different chronic stress levels

The median total score of TICS-9 of the respondents was 25, and the interquartile range was 18 to 27. In Fig. 1, we can intuitively see the proportion of respondents choosing each option in each item of TICS-9. We can see that among the various items of TICS-9, the respondents who choose never, seldom, and sometimes account for a relatively high proportion, while the respondents who choose often and very often account for a relatively low proportion. In addition, based on the threshold of TICS-9, we divided the respondents into four groups: normal, mild, moderate, and severe. Among them, the proportion of resident physicians in the normal group is 19.69%, the proportion of resident physicians in the severe group is 8.66%, and the proportion of resident physicians with mild and moderate chronic stress was 36.22% and 35.43%, respectively Fig. 2.

To explore the difference in chronic stress perception in various socio-demographic variables, we selected the Chi-square test for statistical analysis according to the data type. In the Chi-square test, we merged mild, moderate, and severe chronic stress into the chronic stress group, forming a new classification: normal group and chronic stress group, and conducted the statistical analysis. The statistical results show that the differences in TICS-9 scores in various socio-demographic variables are not statistically significant Table 1.

### Turnover intention

**Fig. 3:**
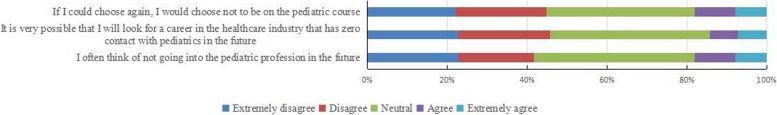
Details of each item on the TIQ

The median of the total score of the TIQ is 9, and the interquartile range is 5 to 9. Among the three questions of the TIQ, 44.88% of the respondents disagreed/extremely disagreed that if they could choose again, they would not choose pediatrics, 45.67% of the respondents disagreed/extremely disagreed that if they had the opportunity, they would choose another career in the healthcare industry with zero contact with pediatrics in the future, and 41.73% of the respondents chose to disagree/extremely disagree in the question “I often think of not going into the pediatric profession in the future” see Fig. 3 for details.

**Fig. 4:**
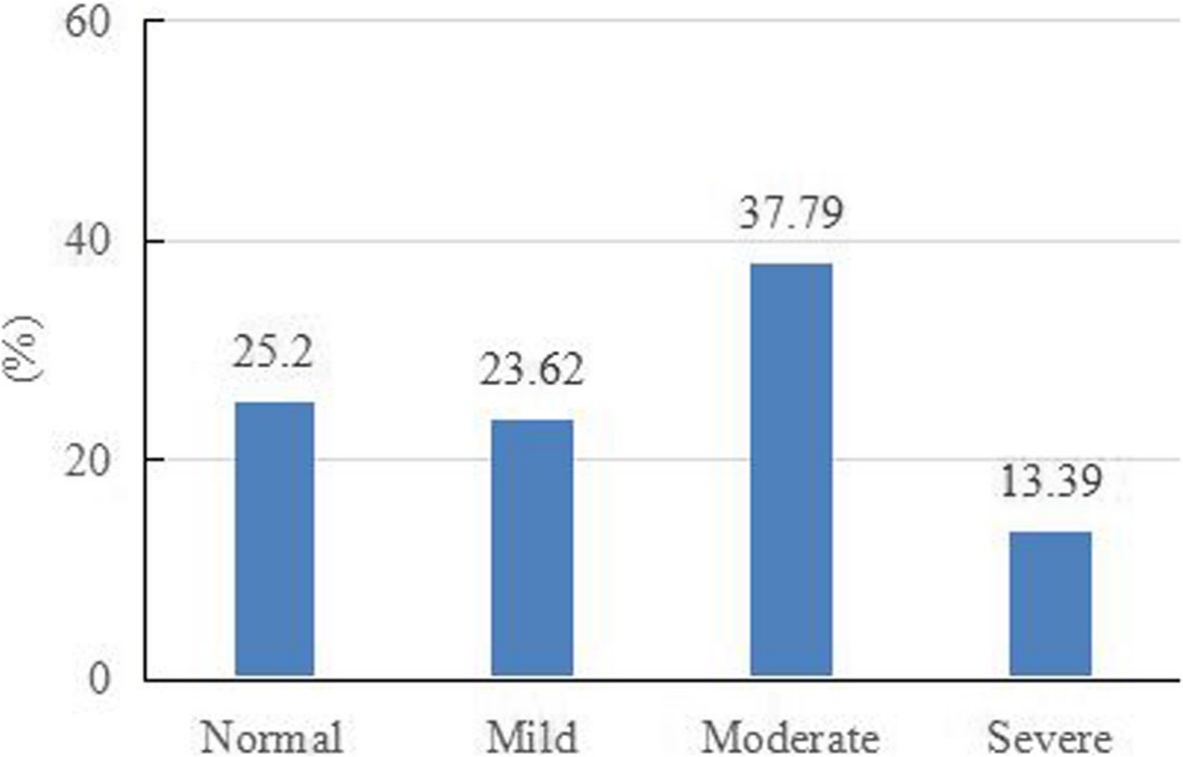
Proportion of respondents with different turnover intention levels

In addition, based on the threshold of TIQ, we divided the respondents into four groups: normal, mild, moderate, and severe. Among them, the proportion of resident physicians in the normal group is 25.2%, the proportion of resident physicians in the severe group is 13.39%, and the proportion of resident physicians with mild and moderate turnover tendencies is 23.62% and 37.79%, respectively Fig. 4. Similarly, we conducted a univariate analysis to explore the differences in turnover intention scores in various socio-demographic variables. The results showed that graduate trainees had lower TIQ scores than non-graduate trainees (*P* = 0.007) Table 2.

**Table 2:** Univariate analysis of scores on the TIQ

Variables		Total N(%)	TIQ		*Chi*^*2*^*-value*	*P-value*
			Normal	Turnover intention		
Gender	Male	27(21.26%)	8(29.63%)	19(70.37%)	0.357	0.550
	Female	100 (78.74%)	24 (24.00%)	76 (76.00%)		
Marital status	Unmarried	88 (69.29%)	25 (28.41%)	63 (71.59%)	1.569	0.210
	Married	39 (30.71%)	7 (17.95%)	32 (82.05%)		
Habitation	Rural	78 (61.42%)	18 (23.08%)	60 (76.92%)	0.482	0.488
	Urban	49 (38.58%)	14 (28.57%)	35 (71.43%)		
Household monthly income (CNY)	≤ 5000	68 (53.54%)	19 (27.94%)	49 (72.06%)	0.585	0.444
	>5000	59 (46.46%)	13 (22.03%)	46 (77.97%)		
Identity	Graduate trainees	79 (62.20%)	25 (31.65%)	54 (68.35%)	4.612	0.032
	Non-graduate trainees	48 (37.80%)	7 (14.58%)	41 (85.42%)		
Year of training	1st	42 (33.07%)	7 (16.67%)	35 (83.33%)	2.569	0.277
	2nd	45 (35.43%)	14 (31.11%)	31 (68.89%)		
	3rd	40 (31.50%)	11 (27.50%)	29 (72.50%)		
Clinical experience before training	No	86 (67.72%)	24 (27.91%)	62 (72.09%)	1.038	0.308
	Yes	41 (32.28%)	8 (19.51%)	33 (80.49%)		

To avoid the influence of confounding factors, we used binary logistic regression analysis to explore the relevant factors affecting turnover intention. We took the turnover intention score as the dependent variable (Normal:3–5; Turnover intention:6–15), and the socio-demographic characteristics and chronic stress perception scores of the trainees as the independent variables to conduct a binary logistic regression analysis. The results are shown in Table 3. Under the control of other conditions, the turnover intention will decrease with each increase in age (OR = 0.772, *P* = 0.042); Compared with graduate trainees, the turnover intention of non-graduate trainees is higher (OR = 8.648, *P* = 0.021); Compared with resident physicians in the normal chronic stress group, resident physicians in the mild, moderate, and severe chronic stress groups have a higher turnover intention, which is 6.983 times, 44.049 times, and 46.141 times, respectively. At the same time, the test results of Omnibus tests of model coefficients and the Hosmer–Lemeshow test showed that our binary logistic regression is reliable Table 3.

**Table 3:** Binary logistic regression analysis of turnover intention

Variables		OR	95%CI		Sig
			lower limit	upper limit	
Gender	Male*				
	Female	2.069	0.511	8.384	0.308
Age	One year increased	0.772	0.602	0.990	0.042
Marital status	Unmarried*				
	Married	2.044	0.419	9.981	0.377
Habitation	Rural*				
	Urban	0.617	0.201	1.896	0.399
Household monthly income (CNY)	≤ 5000*				
	>5000	2.063	0.686	6.203	0.197
Identity	Graduate trainees*				
	Non-graduate trainees	8.648	1.390	53.819	0.021
Year of training	2021*				
	2020	0.811	0.211	3.123	0.761
	2019	0.404	0.086	1.905	0.252
Clinical experience before training	No*				
	Yes	1.032	0.201	5.292	0.970
Chronic stress	Normal*				
	Mild	6.983	1.975	24.691	0.003
	Moderate	44.049	8.433	230.072	<0.001
	Severe	46.141	3.520	604.791	0.004

Omnibus Tests of Model Coefficients: chi-square = 45.819, *p*-value<0.001;

Hosmer–Lemeshowtest: chi-square = 10.598, *p*-value = 0.226;

Nagelkerke R^2^ = 0.448

Notes: * means reference

## Discussion

In this study, we conducted a cross-sectional survey by distributing questionnaires to evaluate the chronic stress and turnover intention of resident physicians after experiencing COVID-19. The results showed that 36.22% and 35.43% of resident physicians reported mild and moderate chronic stress, and 8.60% of resident physicians reported severe chronic stress. This means that 80.31% of resident physicians have varying degrees of stress perception, and only 19.69% of resident physicians are in the normal group.

Compared to medical students or other healthcare workers during COVID-19, the reported proportion of resident physician stress in this study is not optimistic. A previous meta-analysis containing 201 studies showed that during COVID-19, the prevalence of stress among medical students was 34%, 95% CI (27% -42%) [47]. A previous meta-analysis with a sample size of 16,235 showed that the prevalence of stress among healthcare workers during the COVID-19 was 40.3%, 95% CI (31.4% – 50.0%), and the prevalence of stress among healthcare workers in China was 44.2%, 95% CI (30.9%-58.4%) [48]. Finally, a meta-analysis conducted on frontline healthcare workers caring for COVID-19 patients showed that the overall prevalence of stress among frontline healthcare workers was 45% [49].

Similarly, compared to studies conducted before COVID-19, the proportion of resident physician stress reported in this study is also worrying. A survey conducted in Brazil in 2017 found that 55.0% of resident physicians are under normal stress, 12.4% are under low stress, 14.8% are under mild stress, 9.3% are under severe stress, and 8.4% are under extremely severe stress [50]. Another study found that the prevalence of stress among resident physicians is as high as 70.4%, including mild stress (27.4%), moderate stress (20.4%), and severe stress (22.6%) [51].

However, it is not difficult to understand the high proportion of stress perception among resident physicians. The outbreak of COVID-19 has been described as an important source of chronic stress, and medical workers bear the brunt of great pressure during COVID-19 [8, 27]. For resident physicians, the pandemic has also led to direct changes in work and professional training [52]. As described in the introduction, resident physicians have the dual status of formal doctors and medical students, and the difficulties they face are more complex and difficult to overcome [21]. First of all, during the public health crisis, resident physicians still need to assume a lot of responsibilities in medical clinical work, which will undoubtedly increase their risk of exposure to viruses [22, 53]. Secondly, the resident plan was severely impacted, and the planned schedule that should have taken effect was seriously affected [54]; In addition, the significant reduction in the number of patients in the inpatient service limits the education of trainees, and the disease pathological diversity exposed to trainees is significantly reduced [55]; Finally, the transformation of education mode is also a challenge. To avoid unnecessary contact, traditional offline teaching has been changed to an online platform. These changes have brought unique challenges to practice-based clinical learning [56].

As for turnover intention, this study reported relatively high turnover intention results, with only 25.20% of resident physicians in the normal group. About 74.80% of resident physicians have varying degrees of turnover intention, including 23.62% being mild, 37.79% being moderate, and 13.39% being severe. This result is higher than the research conducted in another tertiary hospital in China before COVID-19 (37.8%) [57]. This is similar to the results of other researchers. Previous studies also found that outbreaks of MERS and COVID-19 increased the turnover intention of medical workers [15, 58].

This can be explained by stress. Previous researchers have found that stress related to COVID-19 can induce turnover intention among healthcare workers [59]. Moreover, stress can not only have a substantial direct impact on turnover intention but also exert its indirect effect through job satisfaction [60]. In the context of the COVID-19, high infection risk, heavy workload, shortage of medical supplies, fear of COVID-19, and poor working conditions may adversely affect the job satisfaction of medical workers [14, 61–63], and then affect turnover intention [64].

Interestingly, when we conducted a univariate analysis of turnover intention, we found that TIQ scores showed statistical differences only in identity variable. However, as we further conducted a binary logistic regression analysis, we found that age (OR = 0.772, *P* = 0.042), identity (OR = 8.648, *P* = 0.021), and chronic stress level (mild: OR = 6.938, *P* = 0.003; moderate: OR = 44.049, *P* < *0.003;* severe: OR = 46.141, *P* = 0.004) could significantly affect turnover intention, excluding the interference of other factors. We speculate that the results of univariate analysis are not sensitive due to the co-effect of other confounders. Similar to our results, in the study of other researchers in the medical industry, we also found that age hurts turnover intention [65–67]. It may be due to higher expectations of working conditions among young people, and differences in actual conditions may lead to psychological imbalance and encourage them to resign [68]. As for the impact of identity on resident physicians' turnover intention, sunk cost and choice may explain the reason [34]. The improvement in education level means that students should pay more effort, and more energy, and face greater pressure in exchange for progress on the professional road. Especially in China, being a medical graduate student means that you have to make great efforts to learn medical knowledge to ensure that you perform better than other competitors in the postgraduate entrance examination. After making a lot of effort, the resignation of graduate students means that they will waste their previous potential efforts. In addition, in the postgraduate entrance examination, students have the right to choose their favorite majors. Under the combined action of these factors, our result is not difficult to understand. In addition, our study found that chronic stress can positively affect the turnover intention level of resident physicians. However, when we looked for relevant literature, we found that few studies paid precise attention to the relationship between chronic stress and turnover intention, and they paid more attention to the positive impact of occupational stress on turnover intention [69–71]. Our study can serve as important supplementary evidence of the relationship between chronic stress and turnover intention.

Based on the data in our research report, we strongly suggest that relevant departments pay more attention to the group of resident physicians and implement strong measures to deal with the difficulties faced by resident physicians. Firstly, it is necessary to ensure a reasonable working schedule for resident physicians. Previous researchers reported that the average weekly working hours of resident physicians in China were 83.28 ± 8.80 h [72], which is significantly higher than the data reported by researchers in other countries (66.24 ± 14.97 h) [73]. Long working hours and heavy workloads can lead to job burnout [72], further leading to medical accidents or lower quality of care [74]. The second is to ensure the welfare of resident physicians when they participate in training. During the training period, resident physicians undertake a lot of clinical work but only a small amount of welfare benefits [21, 22]. Researchers have found that the financial burden faced by resident physicians can significantly increase their pressure, and even lead to resident physicians quitting the training program halfway [75]. Finally, support from others can significantly alleviate the negative impact from the outside, and the support of teachers, family members, and friends is also a very important help for resident physicians [46]. Of course, not only resident physicians, we also call on everyone to pay attention to the entire medical system, because resident physicians are only a part of it. In the current economic and social system, how to protect the rights and interests of medical workers, and reduce the pressure and turnover intention of medical workers, is a question that we should consider.

In addition, we have to mention the limitations of this study. First of all, this is a single-center, cross-sectional study, and the respondents are relatively young in age (median: 27). Other researchers should pay attention to the differences in samples when applying this study. Secondly, we issue the questionnaire through the resident physicians' competent department, which may lead to some resident physicians' difficulties in fully expressing their difficulties due to some concerns. In this article, most of the questions in TICS-9 and TIQ are focused on neutral answers, which may come from the implicitness of Chinese people or the difficulty in fully expressing oneself in the face of supervisors. This point needs to be noted. Although there are some limitations in this study, we still believe that it is a reliable study, because we have a high response rate. For potential concerns of respondents, we have adopted more detailed criteria in grouping, dividing chronic stress and turnover intention into four groups: normal, mild, moderate, and severe for subsequent statistical analysis. Finally, we hope that future researchers can pay more attention to the group of resident physicians and solve the difficulties faced by resident physicians.

## Conclusion

In conclusion, this study focuses on the chronic stress level and possible turnover intention of resident physicians after experiencing COVID-19. Among 127 respondents, 80.31% of resident physicians experienced varying degrees of chronic stress (mild: 36.22%, moderate: 35.43%, severe: 8.66%), and 74.80% of resident physicians showed varying degrees of turnover intention (mild: 23.62%, moderate: 37.79%, severe: 13.39%). Moreover, age (OR = 0.772, *P* = 0.042), identity (OR = 8.648, *P* = 0.021), and chronic stress levels (mild: OR = 6.938, *P* = 0.003; moderate: OR = 44.049, *P* < 0.003; severe: OR = 46.141, *P* = 0.004) can significantly affect turnover intention. We suggest that relevant departments should pay more attention to the resident physicians and formulate targeted measures to ensure the sustainability of health human resources. Meanwhile, as this study is a single-center, cross-sectional study, we suggest that future researchers conduct multicenter, longitudinal studies on this basis to more accurately assess the chronic stress and turnover intention levels of resident physicians.

## Data Availability

The original data supporting the conclusion of this study can be requested from the corresponding author through reasonable request.

## Abbreviations

TICS-9: The nine-item short form of the Trier Inventory for Chronic Stress Scale
TICS-57: The original Trier Inventory for Chronic Stress Scale with 57 items
TIQ: The Turnover Intention Questionnaire
