# Correction to: Chronic stress and turnover intention of resident physicians after experiencing COVID-19

**DOI:** 10.1186/s12909-023-04783-3

**Published:** 2023-10-25

**Authors:** Qingwen Jia, Yi Qu, Huisheng Huo, Hongxia Yin, Meijun Jiang, Dianping You

**Affiliations:** 1 Organization and Personnel Department, Children’s Hospital of Hebei Province, Shijiazhuang, China; 2 Scientific research division, Children’s Hospital of Hebei Province, Shijiazhuang, China; 3 Human resources department, Affiliated hospital of north Sichuan medical college, Nanchong, China; 4 Graduate School, Hebei Medical University, Shijiazhuang, China; 5 Party and Government Integrated Office, Children’s Hospital of Hebei Province, Shijiazhuang, China

**Correction to: BMC Medical Education (2023) 23:707**

10.1186/s12909-023-04681-8

Following publication of the original article [1], the author Meijun Jiang has been incorrectly affiliated.

Incorrect affiliation is: Human resources department, Affiliated hospital of north Sichuan medical college, Nanchong, China.

Correct affiliation is: Graduate School, Hebei Medical University, Shijiazhuang, China.

The original article has been corrected.
